# Retrospective investigation of antibodies against chikungunya virus (CHIKV) in serum from febrile patients in Mozambique, 2009–2015: Implications for its prevention and control

**DOI:** 10.1371/journal.pone.0213941

**Published:** 2019-03-21

**Authors:** Virgilio Santo Antonio, Nádia Alves Amade, Argentina Felisbela Muianga, Sadia Ali, Vanessa Monteiro, Flora Mula, Imelda Chelene, John Oludele, Inocêncio Chongo, Américo José, Orvalho Augusto, Eduardo Samo Gudo

**Affiliations:** 1 Virus Isolation Laboratory, National Institute of Health, Maputo, Mozambique; 2 Polana Caniço Research and Training Center, National Institute of Health, Maputo, Mozambique; 3 Department of Microbiology, Faculty of Medicine, Eduardo Mondlane University, Maputo, Mozambique; Universidad Nacional Mayor de San Marcos, PERU

## Abstract

**Introduction:**

Longitudinal data and trends about chikungunya virus (CHIKV) are critical for its control, however in Mozambique very few studies were conducted over 5 decades, between 1957 and 2013. In this study, we retrospectively investigated the occurrence, geographical distribution and trend of anti-CHIKV antibodies between 2009 and 2015 in Mozambique using serum samples from febrile patients.

**Methods:**

A total of 895 serum samples collected from febrile patients for measles and rubella surveillance between 2009 and 2015 in 127 districts of Mozambique were retrospectively tested for IgM and IgG antibodies against CHIKV using a commercially available ELISA.

**Results:**

The median age of patients was 2 years (IQR: 1–5 years) and 44.2% (395/895) of them were female. We found that 54 (6.0%) of samples were positive for anti-IgM chikungunya, and 160 (17.9%) were positive for anti-CHIKV IgG. Antibodies against CHIKV (IgM and IgG) were identified in serum throughout 2009 to 2015. While frequency of IgG antibodies was significantly higher in 2015 as compared to other years, frequency of IgM antibodies was homogeneous between 2009 and 2015. Antibodies against CHIKV were reported in all provinces and in 84 (66.1%) of the districts studied. Frequency of IgM and IgG antibodies was not significantly similar between age groups.

**Conclusion:**

This is the largest and longest serological screening of antibodies against CHIKV in febrile patients in Mozambique and findings from this study suggest that Mozambicans from all over the country have been silently exposed to CHIKV for several years.

## Introduction

Chikungunya virus (CHIKV) is a mosquito-borne virus belonging to the *alphavirus* genus, of the *Togaviridae* family [1, 2]. Over the last two decades, CHIKV has expanded at an alarming pace worldwide, and currently is the second most rapidly spreading arbovirus, after dengue virus (DENV) [3–5]. The virus is transmitted mostly by the bites of *Aedes aegypti* and *albopictus* mosquitoes in tropical and sub-tropical places [6, 7]. CHIKV was described for the first time during an outbreak that was reported in southern Tanzania and northern Mozambique in 1952 [8, 9] and over the last decades has expanded to Southeast Asia, South America, Europe and the Pacific islands [3, 4, 7, 10]. However, for many years, little public health importance was given to CHIKV, until 2005 when a severe outbreak occurred in Reunion Island, causing thousands of cases and hundreds of deaths [11, 12].

Despite the fact that CHIKV tends to cause a self-limiting disease, infection by this virus can cause a chronic and debilitating arthralgia. Haemorrhagic symptoms, encephalitis, coma and deaths have also been reported occasionally [2, 13, 14]. There is no vaccine for chikungunya and treatment is palliative [2, 15].

In sub-Saharan Africa, the virus is often neglected and only a limited number of cases are annually reported, but the risk of CHIKV in the continent is expected to be high, due to its suitability to *Aedes* [5]. The belief is that CHIKV has been circulating silently in the region. The main reasons for under reporting of chikungunya are: lack of awareness by clinicians, weak surveillance systems, similarity of its symptoms to other frequently occurring infections and lack of diagnostic capacity for CHIKV [16].

In Mozambique, the first description of CHIKV was made by Kokernot et al in a sero epidemiological study conducted in 1957. In this study, Kokernot et al found neutralizing antibodies against CHIKV among non-febrile indigenous people living in 29 districts from throughout the country [17]. Additional, but smaller serological investigations conducted in 1971–1973 [18] and 1987 [19] also found serological evidence of CHIKV infections in Mozambique.

However, for several decades the virus remained ignored until 2013, when our group found antibodies against CHIKV in febrile patients in Maputo city [16, 20], followed by description of a case of severe CHIKV infection in northern Mozambique in 2014 [14] and subsequent identification of antibodies against the virus in febrile patients in Quelimane city, in the Central region of the country during an investigation of a potential CHIKV outbreak in 2016 [21].

Longitudinal data and trends about CHIKV are critical for its control and prevention, for this reason there is a great interest in understanding whether CHIKV was circulating in Mozambique prior to 2013. For this purpose, serum samples from febrile patients collected between 2009 and 2015 were retrospectively screened for anti-CHIKV antibodies.

## Materials and methods

### Study design, settings and samples

**Fig 1:**
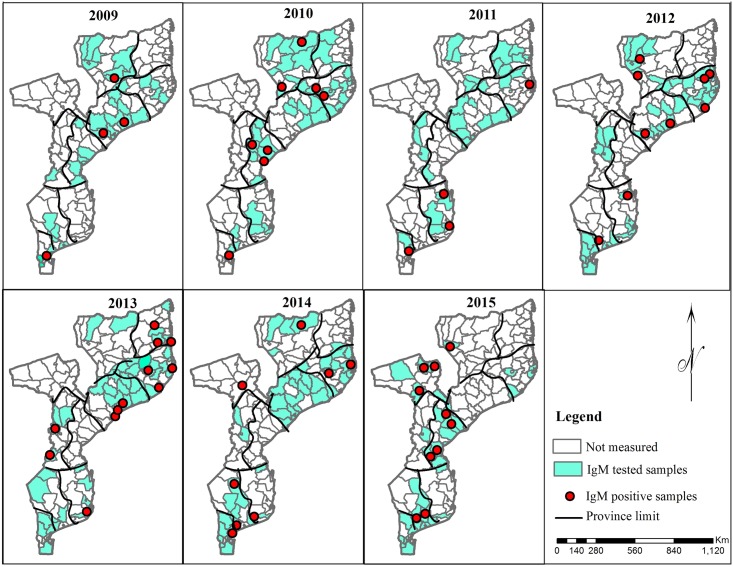
Districts where samples were collected and anti-CHIKV IgM antibodies were detected between 2009 and 2015. Software: ArcGIS 10.2 Software (ESRI Inc, Redlands, CA). Base layers source (Shapefile): Direcção Nacional de Geografia e Cadastro.

**Fig 2:**
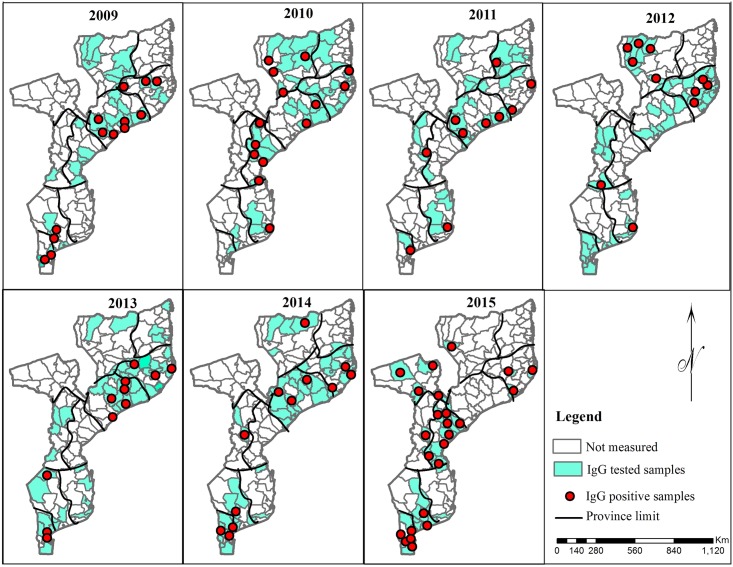
Districts where samples were collected and anti-CHIKV IgG antibodies were detected between 2009 and 2015.

We conducted a retrospective study to investigate presence of antibodies against CHIKV between 2009 and 2015 in Mozambique. We retrieved and screened serum samples being stored in the biobank of the National Institute of Health in Mozambique. This biobank comprises of serum samples collected as part of the routine case-based surveillance for measles in Mozambique between 2009 and 2015. Samples were collected from throughout the country as shown in Figs 1 and 2. Measles surveillance follows World Health Organization guidelines, in which a suspected measles case was defined as a patient with a fever and one of the following symptoms: rash and cough, coryza or conjunctivitis [22]. Whole blood was collected from all suspected cases and serum was separated at the local health facility and shipped to reference laboratory at National Institute of Health for centralized testing by ELISA. In addition, from each suspected patient, a case investigation form was filled and shipped together with the serum to the reference laboratory. The serum biobank was stored at -20°C at the reference laboratory. We selected this biobank because fever and rash are common presentations of chikungunya fever [2].

Mozambique is situated in the southeast coast of Africa and has a tropical climate. The total population of Mozambique is 28.9 million [23]. The country is administratively divided into three regions (north, central and south), 11 provinces and 152 districts. The total surface area of Mozambique is 801,590 km^2^ and the extension of coastline is 2,500 km.

### Eligibility criteria

In this study, only samples collected between 2009 and 2015 with measles and rubella negative results and collected between day 3 and day 7 of onset of fever were selected (See S1 Table). All samples with insufficient serum volume, with inappropriate labelling, and without demographic data in the database were excluded. In this study, only samples collected between 3 and 7 days after the onset of fever from patients with measles and rubella negative results were selected (S1 Table) because in the beginning of the study besides serology we also intended to perform PCR. However, PCR was not performed in this study because quality of sample was not appropriate for PCR.

### Ethics statement

The protocol of this study was approved by the National Bioethics Committee for Health from Mozambique.

### Demographic information

Demographic information, namely age, sex and district was obtained from the electronic database available at the Serology laboratory of the National Institute of Health. The variables retrieved from this database were: age, gender, district, province and year.

### Laboratory testing

Serum samples (n = 895) were screened for anti-CHIKV antibodies (IgM and IgG) using a commercially available ELISA (Euroimmune Lübeck, Germany) at Virus Isolation Laboratory (VIL) in Maputo, Mozambique, following the manufacturer’s instructions. Both IgM and IgG antibodies against CHIKV were measured in order to assess serological evidence of recent infection (IgM) and prior exposure (IgG) to CHIKV.

### Statistical analysis

Data was analysed using Stata version 14.0 (College Station, Texas: StataCorp, USA, 2015). For continuous variables, we calculated medians and interquartile ranges (IQR). For categorical variables, we calculated absolute frequencies and proportions. Binomial exact 95% confidence intervals (CI) were used to report uncertainty around proportions. Log-binomial regressions were used to estimate the proportion-ratio and p-value. The variable region was defined using the province variable. In this context, Cabo Delgado, Nampula and Nissa provinces belonged to northern region. Zambézia, Tete, Sofala and Manica provinces belonged to central region. Maputo city, Maputo province and Inhambane provinces belonged to southern region.

## Results

### General characteristics of patients

The median age of patients from whom samples were tested for the presence of antibodies against CHIKV (n = 895) was 2.0 years old [interquartile range (IQR: 1–5 years) and range (0–96 years)] and 44.2% (395/895) of them were female.

In terms of age distribution, data from Table 1 shows that most of patients were aged 1–4 years old (463/895; 51.7%), followed by patients aged < 1 years old (198/895; 22.1%) and aged 5–9 years old (149/895; 16.6%). Distribution of patients was similar in the three regions of the country (North, Central and South). Number of serum samples available for testing increased from 2009 to 2015, except for 2010, which did not follow this pattern (see Table 1).

**Table 1:** Demographic characteristics among anti-CHIKV IgM positive patients.

	Suspected cases reported, n (%)	Anti-CHIKV IgM+, n (%)	Anti-CHIKV IgM+ %(95% CI)	Proportion Ratio (95% CI)	p-value
**Total**	**895 (100)**	**54 (100)**	**6.0 (4.6–7.8)**		
**Sex**					
Male	498 (55.8)	35 (64.8)	7.0 (4.9–9.6)	1.00	
Female	395 (44.2)	19 (35.2)	4.8 (2.9–7.4)	0.68 (0.40–1.18)	0.171
**Age category (years)**					0.123
< 1	198 (22.1)	8 (14.8)	4.0 (1.8–7.8)	1.00	
1–4	463 (51.7)	33 (61.1)	7.1 (5.0–9.9)	1.76 (0.83–3.75)	
5–9	149 (16.6)	7 (13.0)	4.7 (1.9–9.4)	1.16 (0.43–3.14)	
10–14	47 (5.3)	1 (1.9)	2.1 (0.1–11.3)	0.53 (0.07–4.11)	
≥ 15	38 (4.2)	5 (9.3)	13.2 (4.4–28.1)	3.26 (1.13–9.42)	
**Regions**					0.511
North	295 (33.0)	19 (35.2)	6.4 (3.9–9.9)	1.40 (0.69–2.83)	
Central	339 (37.9)	23 (42.6)	6.8 (4.3–10.0)	1.48 (0.75–2.91)	
South	261 (29.2)	12 (22.2)	4.6 (2.4–7.9)	1.00	
**Year of onset**					0.184
2009	67 (7.5)	5 (9.3)	7.5 (2.5–16.6)	1.63 (0.58–4.59)	
2010	129 (14.4)	11 (20.4)	8.5 (4.3–14.7)	1.86 (0.81–4.26)	
2011	91 (10.2)	3 (5.6)	3.3 (0.7–9.3)	0.72 (0.20–2.55)	
2012	98 (10.9)	5 (9.3)	5.1 (1.7–11.5)	1.11 (0.39–3.17)	
2013	123 (13.7)	13 (24.1)	10.6 (5.7–17.4)	2.30 (1.04–5.10)	
2014	169 (18.9)	7 (13.0)	4.1 (1.7–8.3)	0.90 (0.35–2.32)	
2015	218 (24.4)	10 (18.5)	4.6 (2.2–8.3)	1.00	

IgM +—measles suspected cases with positive result for anti-CHIKV IgM. CI—Confidence interval. CHIKV—Chikungunya virus

### Demographical characteristics of anti-CHIKV IgM positive patients

Of the 895 samples, 54 (6.0%) were positive for IgM against CHIKV (Table 1). Anti-CHIKV IgM antibodies were noted in all provinces of the country and in 84 (66.1%) of the districts studied (Fig 1).

Frequency of anti-CHIKV IgM antibodies was higher in males (7.0%) as compared to females (4.8%), but the difference did not reach statistical significance.

The median age of IgM-positive patients was 2 years (IQR: 1–4 years) (See Table 1). Frequency and proportion ratio of anti-CHIKV IgM antibodies was not significantly different between age groups, despite there was trend towards higher frequency 13.2 (4.4–28.1) and proportion ratio (3.26; 95% CI; 1.13–9.42) when compared to the reference group (children aged < 1 year old).

The frequency of anti-CHIKV IgM antibodies was slightly higher in the Central region as compared to other regions. The proportion ratio was also slightly higher in this region compared to the reference region, but these differences did not reach statistical significance (p = 0.511).

Data from Table 1 also show slight variations in the frequency of IgM antibodies between 2009 and 2015, which did not follow a specific temporal pattern or trend.

### Demographical characteristics of anti-CHIKV IgG positive patients

Of the 895 samples, 160 (17.9%) were positive for IgG against CHIKV and the median age of IgG-positive patients was 2 years (IQR: 1–4 years). Frequency of IgG antibodies against CHIKV was not significantly different between in males and females (17.3% in males versus 18.7% in females) and between different age categories (p = 0.571).

In term of geographical distribution, data from Table 2 show that frequency of anti-CHIKV IgG antibodies was higher in the Central region of the country [21.8% (CI95%; 17.5%–26.6%)] as compared to Northern [16.3% (IC95%; 12.2%– 21.0%)] and Southern [14.6% (CI95%; 10.5%– 19.4%)] parts of the country. Proportion ratio was also higher in Central region (1.50) as compared to the reference group (South region) (p = 0.050).

**Table 2:** Demographic characteristics and relative risk among anti-CHIKV IgG positive patients.

	Suspected cases reported, n	Anti-CHIKV IgG+, n	Anti-CHIKV IgG+ %(95% CI)	Proportion Ratio (95% CI)	p-value
**Total**	**895**	**160**	**17.9 (15.4–20.5)**		
**Sex**					0.571
Male	498	86 (53.8)	17.3 (14.1–20.9)	1.00	
Female	395	74 (46.3)	18.7 (15.0–22.9)	1.08 (0.82–1.44)	
**Age category (years)**					0.911
< 1	198	34 (21.3)	17.2 (12.2–23.2)	1.00	
1–4	463	87 (54.4)	18.8 (15.3–22.7)	1.09 (0.76–1.57)	
5–9	149	26 (16.3)	17.4 (11.7–24.5)	1.02 (0.64–1.62)	
10–14	47	8 (5.0)	17.0 (7.6–30.8)	0.99 (0.49–2.00)	
≥ 15	38	5 (3.1)	13.2 (4.4–28.1)	0.77 (0.32–1.83)	
**Regions**					0.050
North	295	48 (30.0)	16.3 (12.2–21.0)	1.12 (0.76–1.65)	
Central	339	74 (46.3)	21.8 (17.5–26.6)	1.50 (1.05–2.14)	
South	261	38 (23.8)	14.6 (10.5–19.4)	1.00	
**Year of onset**					< 0.001
2009	67	14 (8.8)	20.9 (11.9–32.6)	0.69 (0.42–1.15)	
2010	129	21 (13.1)	16.3 (10.4–23.8)	0.54 (0.35–0.84)	
2011	91	13 (8.1)	14.3 (7.8–23.2)	0.47 (0.27–0.81)	
2012	98	16 (10.0)	16.3 (9.6–25.2)	0.54 (0.33–0.88)	
2013	123	15 (9.4)	12.2 (7.0–19.3)	0.40 (0.24–0.67)	
2014	169	15 (9.4)	8.9 (5.1–14.2)	0.29 (0.17–0.49)	
2015	218	66 (41.3)	30.3 (24.3–36.8)	1.00	

IgG +—measles suspected cases with positive result for IgM anti-Chikungunya. CI—Confidence interval. CHIKV—Chikungunya virus

Table 2 also shows that frequency of anti-CHIKV IgG antibodies was significantly highest in 2015, [30.3% (CI95%; 24.3%%–36.8%)] as compared to other age groups. The highest proportion ratio was also found in 2015, which was set as the reference year in this analysis (p<0.001).

## Discussion

This study represents the largest serological investigation of CHIKV in serum from febrile patients in Mozambique and found both IgM and IgG antibodies against CHIKV in serum collected from 2009 through 2015. Data from this study strongly suggest that Mozambicans were silently exposed to the virus over the last two decades. However, it’s not yet clear whether CHIKV has been circulating in Mozambique since the 50´s or if this represents a more recent introduction of the virus, but further and smaller serological investigations conducted in 1971–1973 [18] and 1987 [19] also found antibodies against CHIKV in 65%-81% (200–400 sera tested each year) and 12.1% (24/199) of samples tested, respectively. Although the frequency of anti-CHIKV IgM antibodies was not significantly different between 2009 and 2015, our study demonstrated that the frequency of IgG was significantly higher in 2015, which deserves more investigation in order to determine the possible recent occurrence of chikungunya outbreaks. The observation that IgG antibodies was significantly higher ins 2015, but IgM was not significantly different between 2009 and 2015 also need further clarification, but might be explained by the fact the IgG is the accumulation of late acute, convalescent and old infection.

Antibodies against CHIKV were found in all provinces and in more than half of the studied districts. We did not find evidence for a specific pattern of positivity for anti-CHIKV IgM antibodies in Mozambique. This suggests that the virus is widespread throughout the country and corroborates findings from the study conducted by Kokernot et al in 1957 [17]. Altogether, this is an added argument in favor of the hypothesis that the virus has been circulating in the country since 50´s.

Few studies have been conducted in other countries in the vicinity of Mozambique, however the frequency of anti-CHIKV IgM antibodies of 6.0% reported in this study was similar to the one reported in a cross sectional study conducted in Tanzania in 2013 (4.7%) [24], but lower than the frequency found in another study conducted in the same country during 2013–2014 (12.9%) [25]. The frequency of CHIKV IgG antibodies of 17.9% found in Mozambique was higher than frequencies reported in the two studies conducted in Tanzania in 2013 (1.6%) [24] and then in 2013–2014 (3.7%) [25], Potential reasons for these differences may be geographical differences, differences in sampling, and differences in seasonality.

For many years, Mozambique was excluded from the list of countries affected by CHIKV [1, 6] and for this reason, no efforts had been made to establish surveillance systems or programs to improve prevention, diagnostics and treatment for chikungunya. Due to the similarity of symptoms of CHIKV infections with other common febrile illness, we believe that most of cases of chikungunya fever were misdiagnosed as malaria or other common acute febrile infections.

In this study, we found no difference in frequency and proportion ratio of IgM and IgG anti-CHIKV antibodies in males and females, suggesting that recent and historical exposure to CHIKV is similar in both genders.

Most of patients were children, because surveillance of measles and rubella is focused on children. This represents a potential limitation, as only few adults were included in this study. The frequency of anti-CHIKV antibodies was not statistically different in different between age groups. This corroborates finding from studies conducted by Kajeguka, *et al* and Sissoko *et al* who found no differences in the frequency of anti-CHIKV antibodies in different age groups [25, 26]. However, Manimunda *et al* and Taraphdar, *et al*, found that chikungunya was more prevalent in adults as compared to younger people [27, 28].

In regard to geographical distribution, there was a trend towards increase of frequency of IgG antibodies against CHIKV in the Central region of the country. This corroborated finding from a recent study conducted in Quelimane city, situated in the Central region of the country, that found a high frequency of IgG antibodies against CHIKV [21]. Higher prevalence of malaria was also noted in the Central region of the country during the most recent malaria indicator survey [29], suggesting that suitability of this Region for mosquito-borne diseases is higher. Indeed, the high average temperatures and humidity in the central region might be associated with higher density of *Aedes* mosquitos in this region.

We acknowledge a number limitations of our study such as the lack of confirmatory test to all ELISA positive results. Virus neutralization assay was not performed due to a lack of resources, and lack of access to high containment laboratories. However, in a recent and joint publication by our group with Institute of Virology, University of Bonn Medical Centre (Bonn, Germany) we were able to demonstrate a high level of agreement between this anti-CHIKV ELISA and plaque-reduction neutralization tests using samples from Mozambique [30], showing that our ELISA results are reliable. We also acknowledge a limitation related to our sampling, first because using data from passive surveillance for measles may under estimate the incidence of chikungunya, second, case definition for measles may not completely overlap with that to chikungunya and third, majority of enrolled patients into measles and rubella surveillance were children. For all these reasons, our data should be interpreted with caution. However, despite this limitation, our data are very relevant for understanding the geographical spread of the virus and variations in the frequency of chikungunya cases in a scenario in which available data are scarce in Mozambique and the surrounding region.

## Conclusion

We performed the largest serological investigation of CHIKV over the last 50 years in Mozambique and found anti-CHIKV antibodies in serum of febrile individuals all over the country between 2009 to 2015. Our findings suggest that Mozambicans were silently exposed to the virus for decades. Data from this study suggests that CHIKV may have become endemic in Mozambique and should be considered in the differential diagnosis of fever. Data from this study also suggest that policies and interventions to control the disease such as i) vector control activities targeting *Aedes* mosquito, ii) community education and mobilization to alert on the risks and prevention measures, iii) increase of clinical awareness among clinicians, and iv) establishment and expansion of the diagnostic capacity within the laboratory network, are urgently needed.

## Supporting information

S1 Table Information on samples of the measles and rubella surveillance, 2009–2015. (DOCX)

S1 File Minimal dataset. (CSV)
