# Supplementary material for: Retrospective investigation of antibodies against chikungunya virus (CHIKV) in serum from febrile patients in Mozambique, 2009–2015: Implications for its prevention and control
Source: PLoS One. 2019 Mar 21;14(3):e0213941. doi: 10.1371/journal.pone.0213941 (PMC6428254; doi:10.1371/journal.pone.0213941)
Supplement: S1 Table — (DOCX) [file pone.0213941.s001.docx]

**S1 Table.** Information on samples of the measles and rubella surveillance

| Year | Total of suspected cases measles and rubella identified | Total measles or rubella positive samples | Total samples tested for antibodies against CHIKV |
| --- | --- | --- | --- |
| 2009 | 536 | 181 | 67 |
| 2010 | 1550 | 725 | 129 |
| 2011 | 889 | 235 | 91 |
| 2012 | 1358 | 440 | 98 |
| 2013 | 977 | 153 | 123 |
| 2014 | 1372 | 295 | 169 |
| 2015 | 1751 | 395 | 218 |
